# Visualizing primer extension without enzymes

**DOI:** 10.7554/eLife.37926

**Published:** 2018-05-31

**Authors:** John C Chaput

**Affiliations:** 1 Department of Pharmaceutical SciencesUniversity of California, IrvineIrvineUnited States; 2 Department of ChemistryUniversity of California, IrvineIrvineUnited States; 3 Department of Molecular Biology & BiochemistryUniversity of California, IrvineIrvineUnited States

**Keywords:** prebiotic chemistry, RNA, enzymes, evolution, dinucleotide intermediate, primer extension, None

## Abstract

X-ray crystallography has been used to observe the synthesis of RNA in the absence of enzymes with atomic resolution.

**Related research article** Zhang W, Walton T, Li L, Szostak JW. 2018. Crystallographic observation of nonenzymatic RNA primer extension. *eLife* **7**:e36422. doi: 10.7554/eLife.36422

How life on Earth began remains one of the greatest scientific mysteries of our time. According to the phylogenetic record, all forms of life, both extant and extinct, are known to store genetic information in DNA and use proteins as enzymes to catalyze metabolic reactions (Joyce, 2002). However, considerable evidence exists to support the idea that modern life was preceded by simpler forms that were based on RNA rather than DNA (Atkins et al., 2011). This hypothetical period, commonly referred to as the RNA world, represents a time when RNA served as the sole genetic material and RNA enzymes (ribozymes) were used to catalyze reactions within primitive cells.

It is well established that RNA can store information and catalyze reactions, so it is possible to envision how modern life could have evolved from RNA-based life. However, it is less clear how the RNA world came into being. Researchers studying this problem have focused on the transition from chemistry to biology, which can be broken down into a number of individual steps, each with its own set of testable hypotheses (Szostak, 2012). Challenges include finding plausible prebiotic routes to the building blocks of life, discovering a mechanism for their assembly into primitive cells, and demonstrating the emergence of Darwinian behaviors through a process of RNA replication with heritable variation.

Recent research advances include the prebiotic synthesis of nucleotides, which are the building blocks of RNA (Powner et al., 2009), and the synthesis of RNA by RNA polymerase ribozymes (Horning and Joyce, 2016; Wang et al., 2011; Wochner et al., 2011). However, despite this progress, significant gaps remain in our knowledge of the RNA world. One major unanswered question is how RNA synthesis happened before the first appearance of an RNA polymerase ribozyme.

Non-enzymatic chemical synthesis of RNA offers a possible bridge between prebiotic chemistry (the molecules and chemical reactions that lead to the emergence of life) and the RNA world (Joyce, 1987). For over 30 years, researchers have used non-enzymatic, template-directed primer extension reactions to study the process of RNA synthesis in the absence of enzymes (proteins or RNA). This approach was thought to involve the stepwise addition of single nucleotides (also known as monomers) to an RNA primer that was base-paired to an RNA template. However, recent work has shown that the critical intermediate is not the monomer, as had been assumed, but a structure containing two nucleotides – called the dinucleotide intermediate (Walton and Szostak, 2016).

Preliminary structural insights into the functional role of the dinucleotide intermediate were initially obtained by X-ray crystallography using crystals of an RNA primer-template duplex bound to a structural analog of the predicted intermediate (Zhang et al., 2017). However, a full understanding of the process requires information about how the structure of the primer-template complex and the actual intermediate changes over time. Now, in eLife, Jack Szostak of the Massachusetts General Hospital and Harvard Medical School and co-workers – Wen Zhang, Travis Walton, and Li Li – report that they have used time-resolved X-ray crystallography to reveal new details about non-enzymatic RNA synthesis (Zhang et al., 2018).

Zhang et al. first made crystals of the RNA primer-template complex with a non-reactive monomer. Activated monomers were then introduced into the crystal by a process of soaking, and as the activated monomers replaced the non-reactive monomers, primer extension began. The researchers used liquid nitrogen to freeze the samples at different times and X-ray crystallography to determine the structures with atomic resolution. This process provided a sequence of events that included all the steps of non-enzymatic RNA synthesis: the activated nucleotides bind to the template, pairs of nucleotides form the intermediate and, finally, a bond is formed between the RNA primer and the intermediate to extend the primer by one nucleotide – all without the involvement of any enzymes.

The observations made by Zhang et al. are nothing short of amazing and will likely influence the field of non-enzymatic RNA synthesis for years to come. In addition to revealing the reaction mechanism, the structures also explain why the primer reacts more readily with the activated intermediate than a template-bound monomer. Namely, pre-organizing the intermediate on the template reduces the distance between the primer and the adjacent nucleotide, which enables the primer to react more rapidly with the dinucleotide intermediate than it could with an individual monomer. While further questions persist, these findings serve as a monumental achievement and a stepping stone towards understanding the origin of the RNA world and the evolution of life on Earth.
